# Supplementary material for: Conformational Preferences of Pyridone Adenine Dinucleotides from Molecular Dynamics Simulations
Source: Int J Mol Sci. 2022 Oct 6;23(19):11866. doi: 10.3390/ijms231911866 (PMC9570408; doi:10.3390/ijms231911866)
Supplement: Supplementary file 1 [file ijms-23-11866-s001.zip › ijms-1903416-supplementary.pdf]

SUPPLEMENTARY MATERIAL

Conformational Preferences of Pyridone Adenine Dinucleotides from Molecular Dynamics Simulations

David P. Buckley[1], Marie E. Migaud[2,3], and John J. Tanner[1,4,*]

[1]Department of Biochemistry, University of Missouri, Columbia, Missouri 65211, USA

[2]Department of Pharmacology, College of Medicine, University of South Alabama, Mobile, AL 36688, USA

[3]Mitchell Cancer Institute, College of Medicine, University of South Alabama, Mobile, AL 36604, USA

[4]Department of Chemistry, University of Missouri, Columbia, Missouri 65211, USA

Table of Contents

**Figure S1.** Time evolution of the inter-base centroid-centroid distance.	S2
**Figure S2.** Individual centroid-centroid distance distribution for each 100-ns dinucleotide simulation.	S3
**Figure S3.** Plot of the inter-base distance versus adenine glycosidic bond dihedral angle (χ_A).	S4
**Figure S4.** Cluster representations of conformations of NAD^+ and NADH.	S5
**Figure S5.** Selected conformations of NAD(H) and ox-NADs.	S6
**Figure S6.** Cluster representations of conformations ox-NADs.	S7
**Supplementary References**	S8

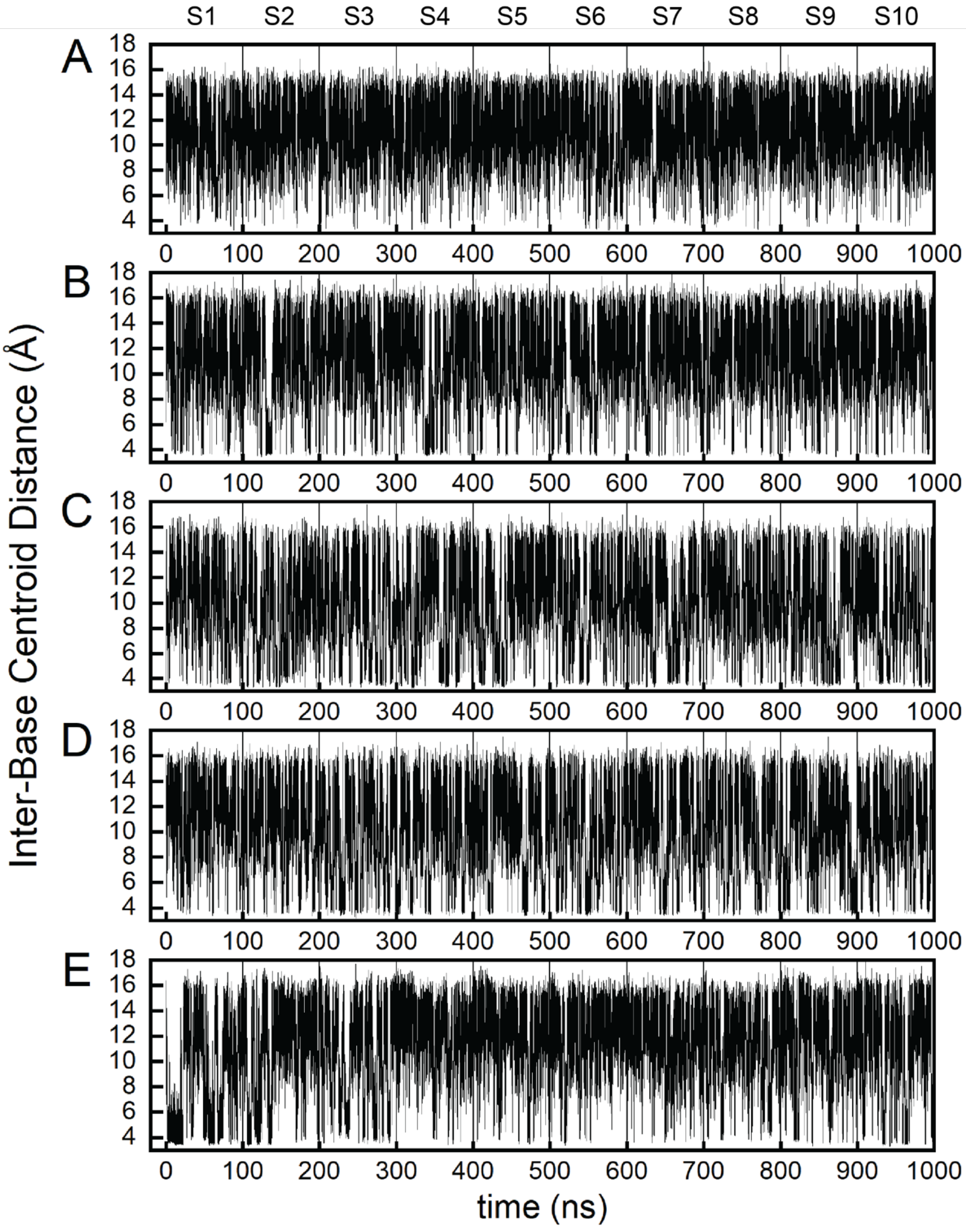

Figure S1. Time evolution of the inter-base centroid-centroid distance for ten 100-ns simulations (S1-S10, separated by vertical lines) of (A) NAD^+, (B) NADH, (C) 2-ox-NAD, (D) 4-ox-NAD, and (E) 6-ox-NAD.

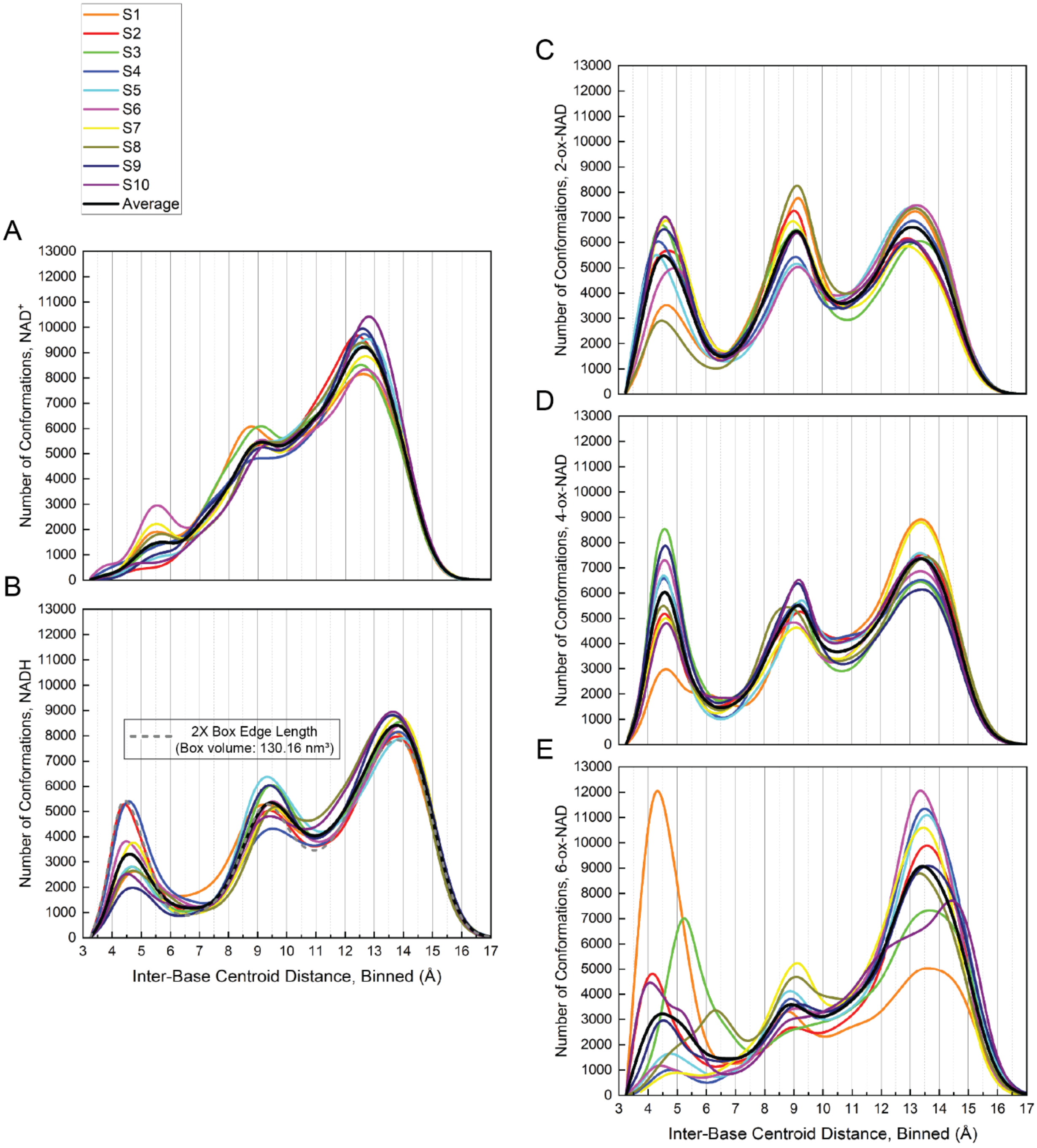

Figure S2. Individual distributions of the centroid-centroid distance between the bases from the ten individual 100-ns simulations (S1-S10) against their average (black) for (A) NAD$^+$, (B) NADH, (C) 2-ox-NAD, (D) 4-ox-NAD, and (E) 6-ox-NAD. The dashed curve in panel B was calculated from a 100 ns simulation of NADH performed with the box volume increased from 130.16 nm^3 to

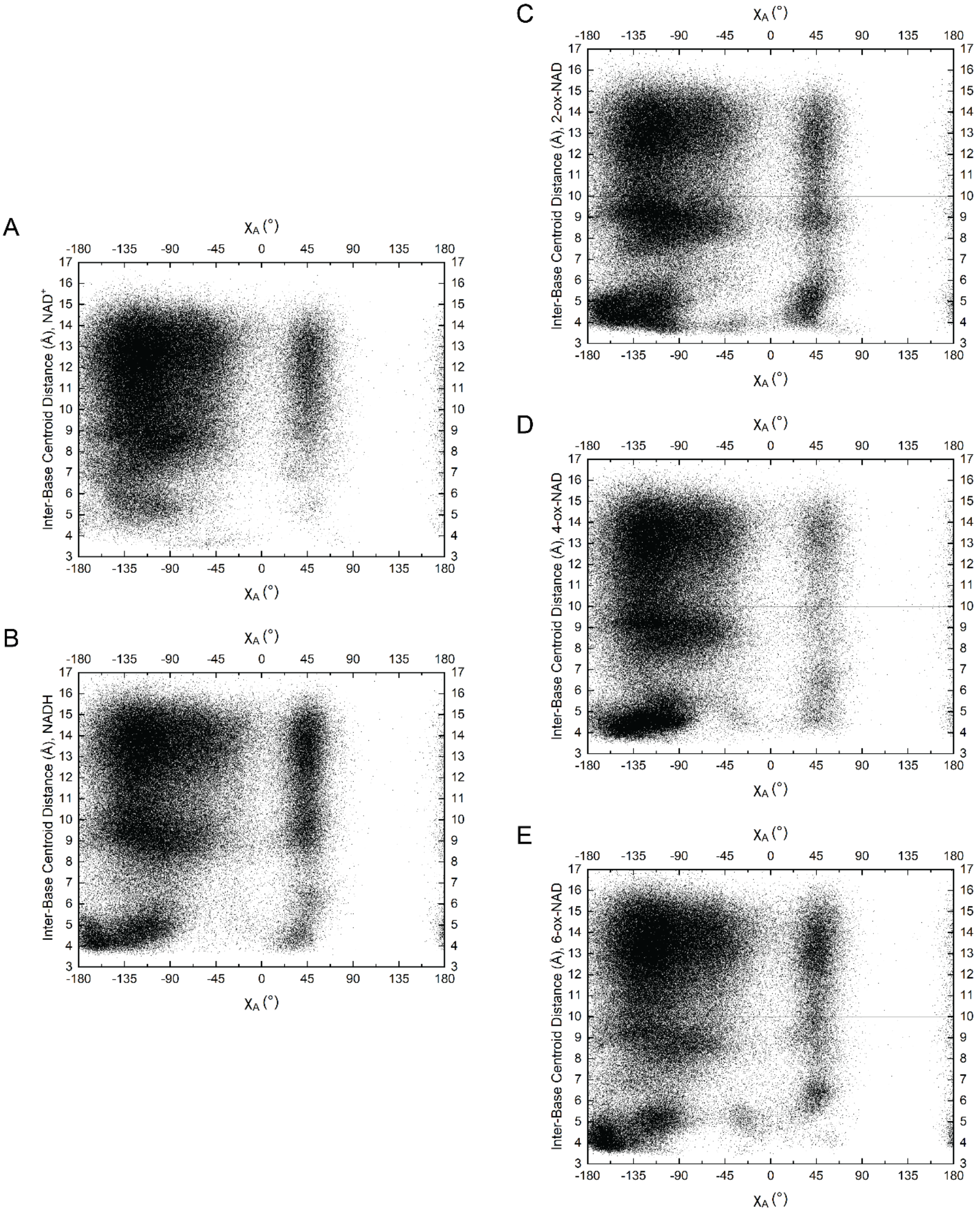

Figure S3. Scatter plot of the inter-base centroid-centroid distance versus adenosine glycosidic bond dihedral angle (χ_A) for (A) NAD^+, (B) NADH, (C) 2-ox-NAD, (D) 4-ox-NAD, and (E) 6-ox-NAD.

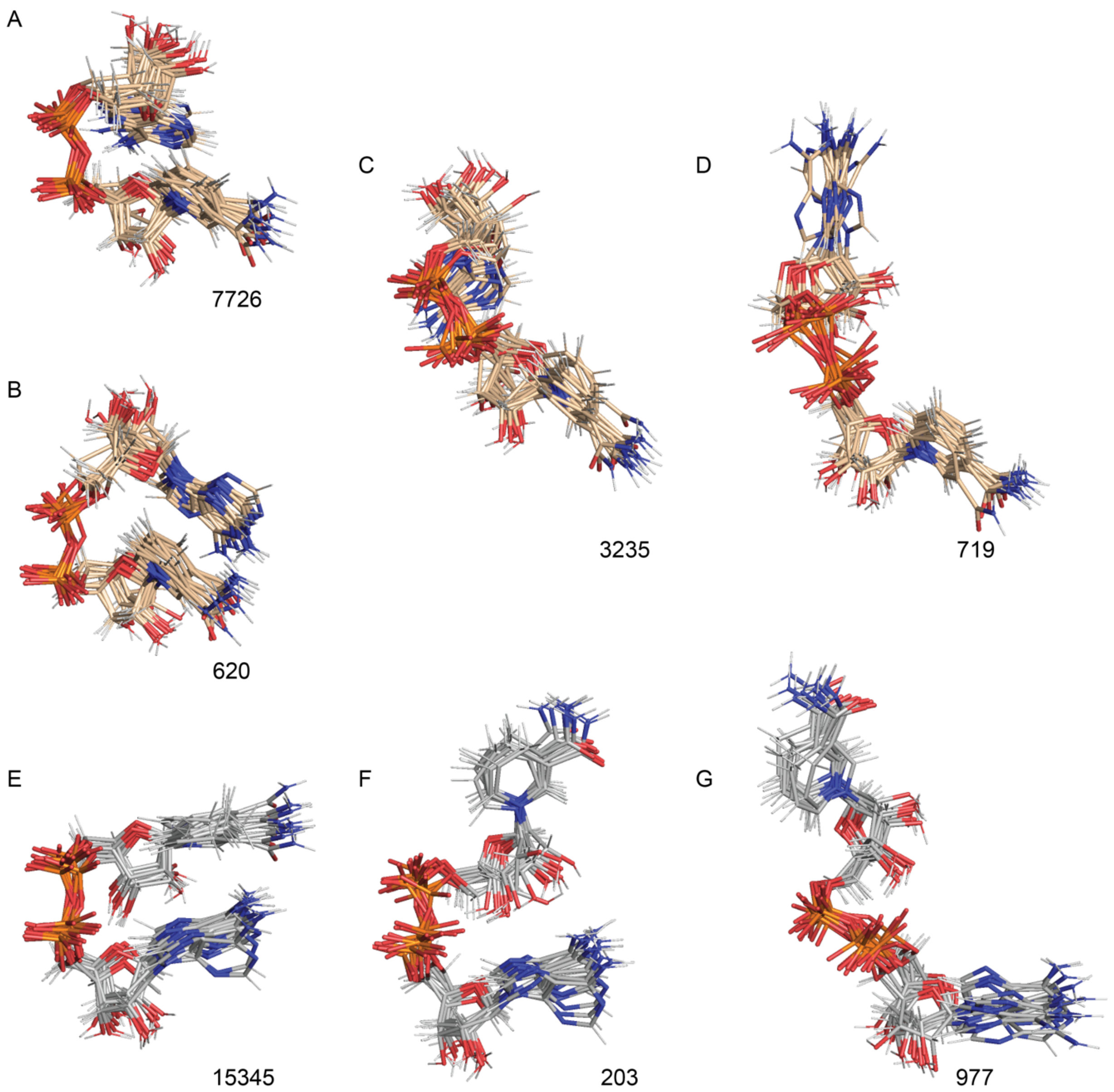

Figure S4. Clusters of conformations of NAD^+ (A-D) and NADH (E-G). Each panel shows 10 conformations selected from conformations within 1.0 Å RMSD of the conformations shown in Fig. 4. The numbers are the total counts of conformations that meet the 1 Å RMSD criterion.

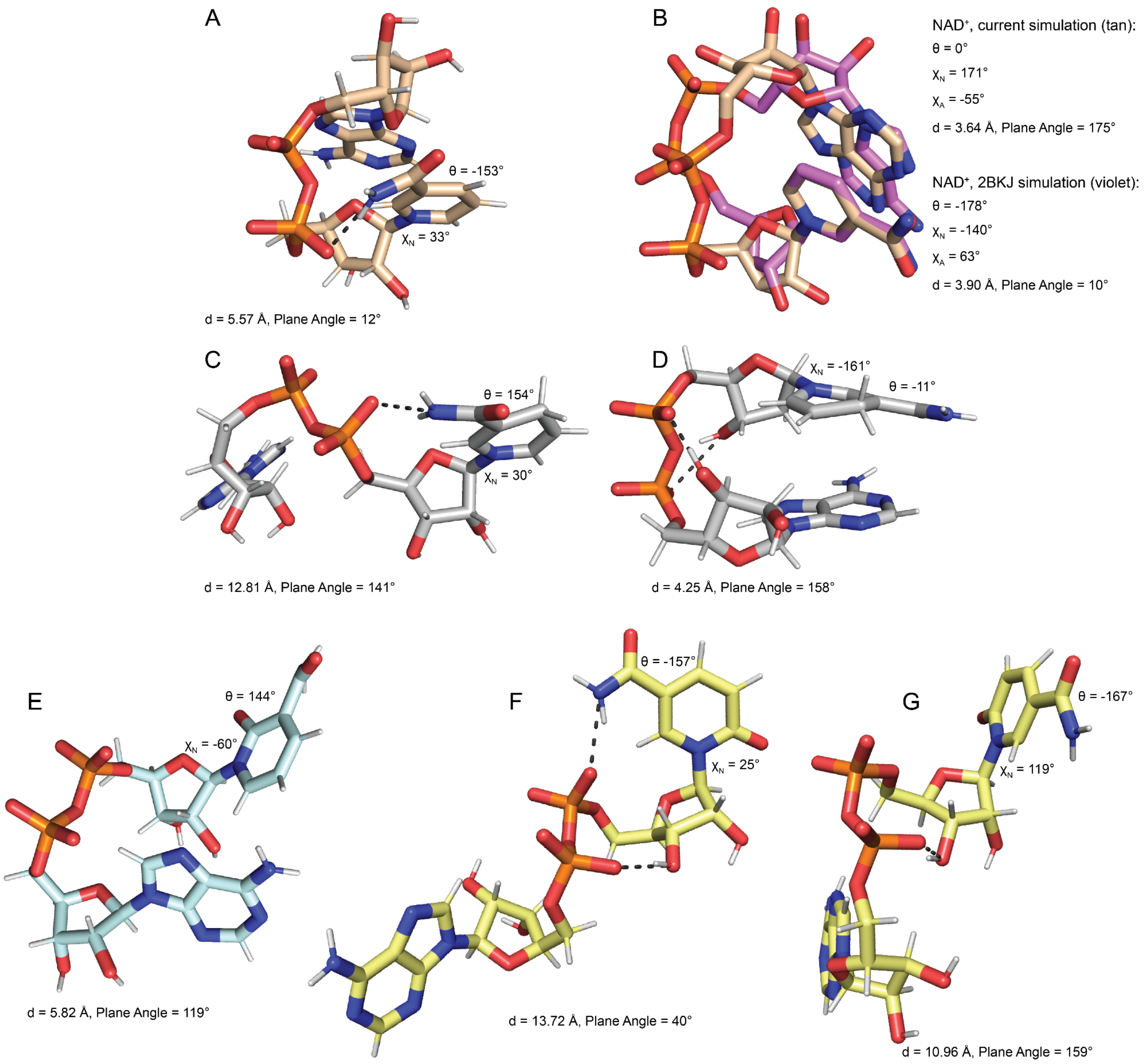

Figure S5. Selected conformations of NAD(H) and ox-NADs. (A) NAD^+ with the nicotinamide ribose *syn*. (B) Overlay of the rare NAD^+ folded conformation from the current simulation (tan) and the starting conformation from a previous simulation of NAD^+ (PDB 2BKJ, chain B, violet) [1]. (C) NADH with the nicotinamide ribose *syn*. (D) Folded NADH with inter-base plane angle near 160°. (E) High-*anti* pyridone ribose conformation of 2-ox-NAD. (F) 6-ox-NAD with the pyridone ribose *syn*. (G) High-*syn* pyridone ribose conformation of 6-ox-NAD. Values of the inter-base centroid distance 'd', plane angle, θ, and χ_N are indicated for each pose. Hydrogen bonds are indicated by dashed black lines between heavy atoms.

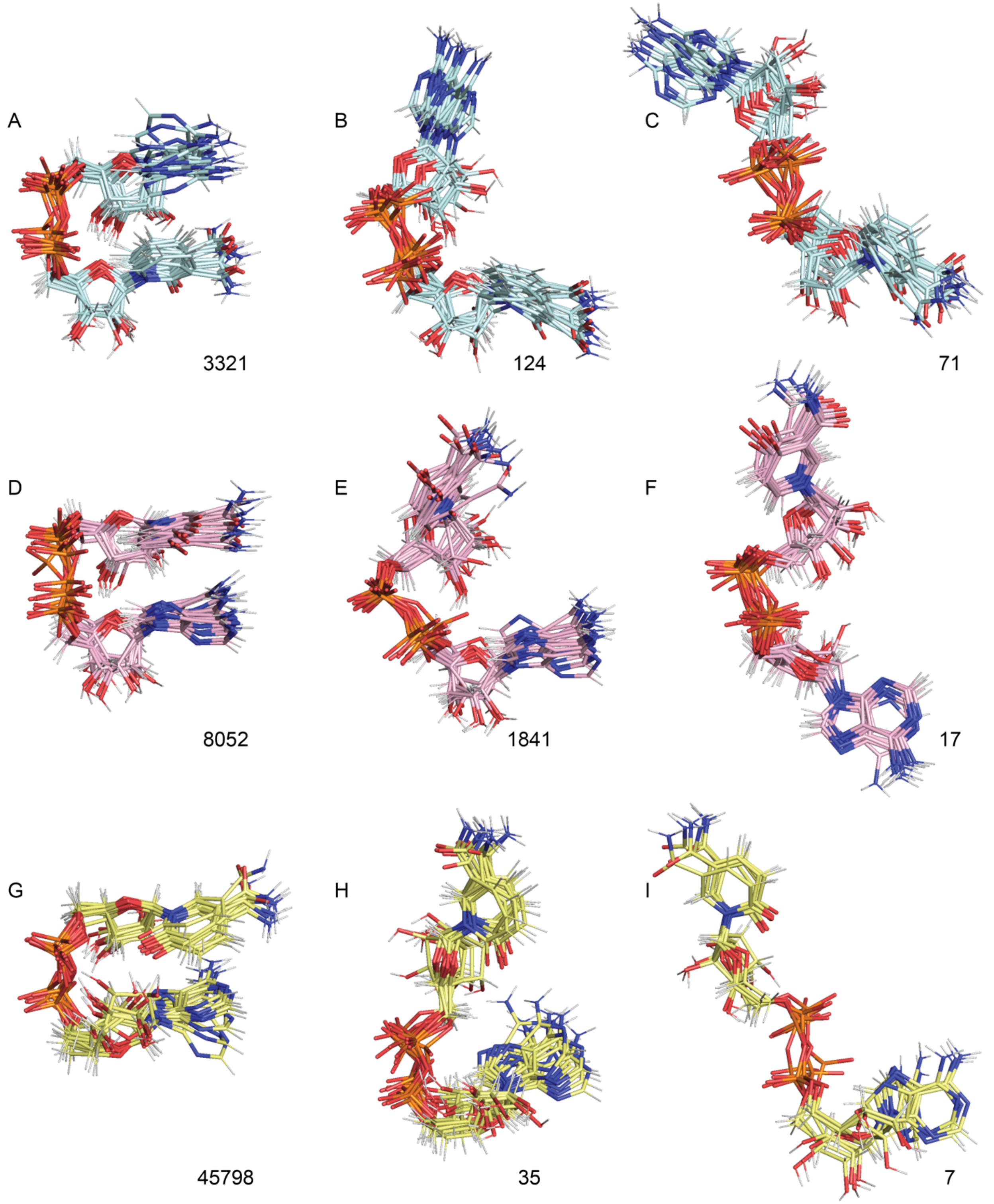

Figure S6. Clusters of conformations of 2-ox-NAD (A-C), 4-ox-NAD (D-F), and 6-ox-NAD (G-I). Each panel shows 10 conformations selected from conformations within 1.0 Å RMSD of the conformations shown in Fig. 6, except for panel I, which contains 7 poses in the cluster. The numbers are the total counts of conformations that meet the 1 Å RMSD criterion.

Supplementary References

[1] P.E. Smith, J.J. Tanner, Molecular dynamics simulations of NAD^+ in solution, J. Amer. Chem. Soc. 121(37) (1999) 8637-8644.
